# The Effects of Tocotrienol-Rich Vitamin E (Tocovid) on Diabetic Neuropathy: A Phase II Randomized Controlled Trial

**DOI:** 10.3390/nu12051522

**Published:** 2020-05-23

**Authors:** Yeek Tat Ng, Sonia Chew Wen Phang, Gerald Chen Jie Tan, En Yng Ng, Nevein Philip Botross Henien, Uma Devi M. Palanisamy, Badariah Ahmad, Khalid Abdul Kadir

**Affiliations:** 1 Jeffrey Cheah School of Medicine and Health Sciences, Monash University Malaysia, Jalan Lagoon Selatan, Bandar Sunway, Subang Jaya 47500, Selangor, Malaysia; soniaphang@gmail.com (S.C.W.P.); gctan73@gmail.com (G.C.J.T.); enyng24@gmail.com (E.Y.N.); nevein.botross@monash.edu (N.P.B.H.); umadevi.palanisamy@monash.edu (U.D.M.P.); badariah.ahmad@monash.edu (B.A.); khalid.kadir@monash.edu (K.A.K.); 2 Tropical Medicine and Biology Platform, School of Science, Monash University Malaysia, Jalan Lagoon Selatan, Bandar Sunway, Subang Jaya 47500, Selangor, Malaysia

**Keywords:** type 2 diabetes mellitus, diabetic peripheral neuropathy, tocotrienol, vitamin E, nerve conduction velocity, nerve conduction study, nerve growth factor (NGF), malondialdehyde (MDA), vascular cell adhesion molecule 1 (VCAM-1), tumor necrosis factor receptor 1 (TNFR-1)

## Abstract

Chronic hyperglycemia increases oxidative stress, activates inflammatory pathways and reduces nerve growth factor (NGF) among diabetic patients, which contribute to development of diabetic peripheral neuropathy (DPN). Tocotrienol-Rich Vitamin E (Tocovid) possesses potent antioxidant and anti-inflammatory properties which are postulated to target these pathogeneses in order to ameliorate DPN. This study aims to evaluate the effects of Tocovid on nerve conduction parameters and serum biomarkers among diabetic patients. This multicenter, prospective, randomized, double-blind, placebo-controlled clinical trial was conducted on 80 eligible participants. The intervention group (*n* = 39) was randomly allocated to receive 200 mg of Tocovid twice a day, and the control group (*n* = 41) received placebo twice a day. At the end of eight weeks, the nerve conduction parameters, as assessed by nerve conduction study, as well as serum biomarkers (NGF, malondialdehyde, vascular cell adhesion molecule 1, tumor necrosis factor receptor 1 and thromboxane B2) were compared between the two groups. Compared to placebo, Tocovid significantly improves the nerve conduction velocities of all nerves (+1.25 m/s, interquartile range [IQR] 3.35, *p* < 0.001, median nerve; +1.60 m/s, IQR 1.80, *p* < 0.001, sural nerve; +0.75 m/s, IQR 2.25, *p* < 0.001, tibial nerve). Meanwhile, the levels of serum NGF were significantly higher in the Tocovid group as compared to placebo at eight weeks post-intervention. Participants receiving Tocovid illustrated highly significant improvement in terms of nerve conduction velocities for all nerves tested after eight weeks of supplementation. In addition, Tocovid supplementation elevated the levels of serum NGF, in which its increase is postulated to reflect enhanced neuronal functions. This novel finding suggests that Tocovid could be a disease-modifying agent targeting serum NGF to improve nerve conduction velocities.

## 1. Introduction

Diabetic peripheral neuropathy (DPN) or distal symmetrical polyneuropathy (DSPN), is the most common diabetic neuropathic syndrome, with a ten-year prevalence of approximately 50% among patients with type 2 diabetes mellitus (T2DM) [1,2]. DPN remains a serious complication of diabetes mellitus which needs to be addressed, as it impairs the quality of life and causes significant morbidity as well as mortality.

Approximately half of the patients affected by DPN are asymptomatic, while the other half will either experience distressing neuropathic pain or present with foot ulceration due to diminished sensation [2,3]. Diabetic foot ulceration due to DPN increases the risk of non-traumatic limb amputation and accounts for a major proportion of hospitalization among all diabetic complications [2,4]. The five-year survival rates after developing diabetic foot ulcers and amputation are 50% and 30% respectively [5].

Chronic hyperglycemia leads to activation of various metabolic pathways which may contribute to DPN. These include oxidative stress, inflammatory pathways, polyol pathway, advanced glycation end products (AGEs) pathway, nerve growth factor (NGF), poly(ADP ribose) polymerase (PARP), hexosamine pathway, protein kinase C (PKC) pathway, and a non-exhaustive list of metabolic pathways [6]. Nevertheless, the mainstay of DPN management remains to focus on symptomatic relief and optimal glycemic control [7], rather than addressing the underlying pathogenetic mechanisms of DPN. Despite the availability of drugs that target the underlying pathophysiology of DPN, efficacies of the disease-modifying agents are variable and some have undesirable side effects [8].

Current perspectives on the pathogenesis of DPN mainly focus on oxidative and inflammatory stress responses which stem from the above-mentioned dysfunctional metabolic pathways [2]. Vitamin E, particularly its tocotrienol isoform, possesses potent antioxidant, anti-inflammatory, and neuroprotective properties [9]. Thus, vitamin E may be one of the disease-modifying agents used to attenuate DPN, although its exact mechanism of action still remains unknown [10]. To date, available evidence of its efficacy is limited and inconclusive; conflicting results were reported with regard to improvements in nerve conduction parameters and relief of neuropathic symptoms [10,11,12,13].

Taking the nature of DPN, which is asymptomatic in half of the affected population, into consideration, this clinical trial aimed to evaluate the effects of tocotrienol-rich vitamin E on patients with T2DM, regardless of whether they have ever experienced any neuropathic symptoms directly attributable to DPN. For instance, neuropathic symptoms affected by carpal tunnel syndrome, vibration-induced neuropathy and cerebral vascular accident were excluded from the study. The outcomes analyzed were nerve conduction parameters as assessed by a nerve conduction study (NCS) as opposed to a scoring system for DPN such as Total Symptom Score (TSS), Neuropathy Symptom Score, Michigan Neuropathy Screening Instrument, and Neuropathy Disability Score. It is crucial to note that NCS provides an objective quantification even in the subclinical stages of DPN progression as compared to the rather subjective scoring system [1,8,14]. In addition, NCS is recommended to be used in DPN-related controlled clinical trials and epidemiological surveys [1,14]. This study also explored the various metabolic pathways of DPN which were postulated to be acted upon by vitamin E as assessed by the serum biomarkers. Serum malondialdehyde (MDA) levels were measured as an indicator of oxidative stress; serum vascular cell adhesion molecule 1 (VCAM-1) and tumor necrosis factor receptor 1 (TNFR-1) reflect the inflammatory activities; serum NGF was measured as a biomarker for neuronal function; serum thromboxane B2 (TXB2) was assessed as elevated production contributes to diabetic microvascular complications [15,16].

The main objective of this clinical trial is to investigate the effects of tocotrienol-rich vitamin E on DPN by means of changes in nerve conduction parameters, including nerve conduction velocity and amplitude of action potential as assessed by NCS. Moreover, this clinical trial aimed to assess the effects of tocotrienol-rich vitamin E on serum biomarkers (NGF, MDA, VCAM-1, TNFR-1 and TXB2), and determine the relationship between these biomarkers and nerve conduction parameters.

## 2. Materials and Methods

### 2.1. Study Design and Participants

This was a multicenter, phase II, prospective, double-blind, randomized, placebo-controlled clinical trial undertaken at three clinical centers in Malaysia. This clinical trial was carried out in accordance with the Declaration of Helsinki as well as terms and conditions set forth in the Malaysian Good Clinical Practice (GCP) guidelines. Ethical approval for the study protocol was granted by Monash University Human Research Ethics Committee (MUHREC) before commencement of the study (project number: 12090). This trial was registered in the Australian New Zealand Clinical Trials Registry (ANZCTR), with the trial registration number ACTRN12619001568101, as well as a Malaysian National Medical Research Register (NMRR) trial ID—NMRR-18-3928-45140.

Study participants were recruited from an existing pool of patients who regularly follow up at Monash University Clinical Research Centers (CRCs) in Bandar Sunway and Johor Bahru, respectively, as well as Thomson Hospital, Kota Damansara. Initially, the patients were prescreened by reviewing their medical records kept at respective clinical sites. Patients who were on hypoglycemic agents (oral, injectable or both) diagnosed with T2DM were identified as potential participants. The diagnosis of T2DM was made based on their fasting blood glucose level or HbA1c according to the American Diabetes Association (ADA) Standards of Medical Care in Diabetes 2019 [17]. Among these potential participants, those aged from 35 to 75 years old were briefly informed about the study over the phone. Interested participants were notified to attend the screening visit in accordance with regular diabetes follow-up visit at the CRCs. The patients were prompted to abstain from any food or drinks (except plain water) for at least eight hours and withhold their morning dose of antihyperglycemic agents before visiting the CRCs for screening. Besides, premenopausal women were advised to only attend the screening visit when they were not having menses.

Written informed consent was obtained from all study participants prior to performing any study-related procedures. Included participants were T2DM patients with optimal glycemic control as assessed by HbA1c of 6% to 9%. In the event that they were hypertensive, their blood pressures should be well controlled below 150/90mmHg. On the other hand, patients who had unstable and severe chronic illnesses (recent acute coronary syndrome, active cancer, liver diseases or any inflammatory disorders), estimated glomerular filtration rates (eGFR) of less than 30 mL/min/1.73 m^2^, or unstable eye diseases, as well as pregnant women, were excluded. Furthermore, patients were excluded if they had consumed any water-soluble supplements such as vitamin B, C and glutathione during the two weeks prior, or any fat-soluble supplements such as vitamin A, D, E and K during the one month beforehand. These exclusion criteria ensured that the outcomes of the clinical trial were solely due to Tocovid and not affected by other vitamins or antioxidants.

### 2.2. Randomization and Masking

A total of 80 eligible participants were recruited and randomly assigned in a 1 to 1 ratio into the double-blinded treatment period for eight weeks. The randomization was done by an independent consultant using Microsoft Excel spreadsheet and was stratified according to gender (male or female), duration of diabetes (median of 15 years) and HbA1c levels (median of 7.95%). Participants who were assigned to the intervention group received 200 mg tocotrienol-rich vitamin E (Tocovid, constituents available in the “Supplementary Materials”) twice daily, whereas the control group was given placebo (tocotrienol-free palm oil capsules) twice daily. Both the Tocovid and placebo capsules were sponsored by ExcelVite Pty Ltd. and manufactured by Hovid Pharmaceuticals Berhad. The capsules were similar in size, shape and color. The identity of the investigational products was kept confidential by ExcelVite, in which they were labelled as Drug B and Drug D. The randomization and allocation remained concealed from all the investigators and participants until the study was completed.

### 2.3. Procedures

During the screening visit, a complete medical history and a thorough physical examination were conducted on the potentially eligible participants. Blood pressure was measured thrice when they were sitting upright and an average of three stable readings was recorded. Anthropometric measurements including weight, height and waist circumference were also obtained. Urine sample was collected from the potentially eligible participants to carry out urine full examination microscopic examination (FEME); a urine pregnancy test was performed for premenopausal female subjects. Subsequently, fasting venous blood samples were collected to assess for HbA1c, fasting blood glucose, renal profile, lipid profile, liver function test, vitamin E levels and serum biomarkers (NGF, MDA, TNFR-1, VCAM-1 and TXB2). A nerve conduction study (NCS) was conducted on the patients to obtain the baseline neurological function. The nerves that were assessed include upper limbs median sensory, lower limbs sural sensory and tibial motor nerves. Electrocardiogram (ECG) was performed as a safety test to ensure fitness of participants. As the research team was also investigating the effects of Tocovid on diabetic retinopathy, a retinal photograph was taken to determine the baseline retinal pathology.

Once the biochemistry results were available, participants were informed over the phone regarding their eligibility to participate in the clinical trial. Eligible participants who fulfilled all the inclusion and exclusion criteria were notified to return to one of the Monash University CRCs for randomization visit and collection of investigational products. Participants were followed up at four weeks to monitor for adherence and adverse events. Anthropometric measurements, blood pressure, capillary fasting blood glucose level and urine FEME were routinely performed in each visit for monitoring purpose. At eight weeks post-intervention, fasting venous blood samples were again collected to carry out the aforementioned biochemical tests. NCS were conducted at baseline and at 8 weeks for primary outcome assessment. ECG and retinal photograph were also done at baseline and end of study.

### 2.4. Outcomes

The primary outcome variable of the study was the mean/median of changes in nerve conduction parameters as assessed by NCS at eight weeks post-intervention compared to baseline between the Tocovid and placebo groups. These were measured in order to determine the effects of Tocovid on peripheral nerves’ function. The secondary outcome variables were the serum biomarkers postulated to be affected in DPN, including NGF, MDA, TNFR-1, VCAM-1, and TXB2 at baseline and end of study visits. Fasting venous vitamin E levels were measured to ensure the adherence of the study participants.

### 2.5. Nerve Conduction Study

The nerve conduction study (NCS) was carried out using MedelecSynergy (VIASYS Healthcare, Philadelphia, PA, USA) based on standardized methodology for NCS [18,19]. The sensory nerve tested were median and sural nerves, whereas the motor nerve tested was tibial nerve. These three nerves were tested bilaterally on all the participants. When conducting the NCS, it was ensured that the skin temperature was maintained above 32 °C, over the upper limbs as well as the lower limbs. The parameters recorded in sensory NCS include onset latency, peak latency, sensory nerve action potential (SNAP), conduction velocity (CV) and peak velocity (PV). On the other hand, distal and proximal onset latencies, compound muscle action potential (CMAP), distance between distal and proximal stimulation point, as well as CV were parameters recorded for motor NCS. Details of the NCS methodology used is available in the “Supplementary Materials”.

To ensure validity of the results, NCS was performed using the same methodology on age-matched volunteers recruited without diabetes mellitus to obtain a group of normal control for comparison of the nerve conduction parameters.

### 2.6. Serum NGF, MDA, TNFR-1, VCAM-1 and TXB2 Levels

The blood samples collected were centrifuged on the same day with Eppendorf Centrifuge 5702R (Hamburg, Germany). The sera were stored frozen at a temperature of −80 °C in Eppendorf Tubes and the detection of serum biomarkers levels were done at the end of the study to minimize inter-assay variability. These tests were measured in duplicates and quantified by colorimetric method using enzyme-linked immunosorbent assay (ELISA) with TECAN Infinite 200 PRO (Zürich, Switzerland) and their respective ELISA kits. The ELISA kits include Elabscience E-EL-H1205 (Houston, TX, USA) for NGF, Elabscience E-EL-0060 (Houston, TX, USA) for MDA, Elabscience E-EL-H0217 (Houston, TX, USA) for TNFR-1, Elabscience E-EL-H5587 (Houston, TX, United States) for VCAM-1 and Elabscience E-EL-H2191 (Houston, TX, USA) for TXB2. All the ELISA kits had intra-assay coefficient variances of 4% and inter-assay coefficient variances of 8%.

### 2.7. Renal Profile, Lipid Profile and Liver Function Tests

Serum samples collected during the study visit were sent to the nationally certified pathology laboratory on the same working day for renal profile, lipid profile and liver function test using Abbott Diagnostics ARCHITECT (Elgin, IL, USA), and the coefficient variances for the tests were generally less than 6%. Renal profile parameters measured were blood urea nitrogen (BUN), serum creatinine and estimated glomerular filtration rate (eGFR); lipid profile parameters measured were total cholesterol and high-density lipoprotein cholesterol (HDL-C); liver function test parameters measured were aspartate transaminase (AST) and alanine transaminase (ALT).

### 2.8. HbA1c Assessment

Blood samples were collected and sent to the nationally certified pathology laboratory on the day of collection for HbA1c measurement using Cobas Integra 400 plus analyzer (Roche Diagnostics, Laval, QC, Canada), with a measuring range of 4.3–18.8% and a coefficient variance of 5%.

### 2.9. Adherence

As study participants were required to return the remaining capsules during each follow-up visit, returned capsule count was used to assess for their adherence. In addition, participants’ adherence was determined by fasting venous vitamin E levels at baseline and eight weeks post-intervention. These levels were measured and analyzed at the end of study to maintain blinding. Plasma tocotrienol levels were measurable at four hours post-consumption of Tocovid using a validated-high performance liquid chromatography (HPLC) technique by Che H et al. [20], but not at the fasting state (more than 12 h after Tocovid consumption). Due to inability to measure plasma tocotrienol levels at four hours post-consumption, levels of serum α-tocopherol (third highest constituent of Tocovid, after γ-tocotrienol and α-tocotrienol), with a relatively higher elimination half-life compared to tocotrienol [21], were measured using the validated HPLC method by Liu Z et al. [22], and the values were corrected for lipids [23].

### 2.10. Statistical Analysis

The sample size was calculated based on the sural nerve conduction velocity among diabetic patients which was obtained from a study published by Dunnigan SK et al. [24]. According to recommended sample size calculation for clinical trials, in order to obtain an α value of 0.05 and power of 0.90, with 5% difference in increment of sural nerve conduction velocity between the two arms of the clinical trial, the sample size required in each arm is 36 participants. Taking into consideration that each group might have a 5% dropout rate, the minimum sample size in each arm was determined to be 38, yielding a total number of 76 participants.

All the statistical analyses of the data were performed using the IBM SPSS Statistics v.25 (Armonk, NY, USA) database. Per protocol analyses were performed for this clinical trial. Continuous variables were assessed for normality of data distribution using the Kolmogorov–Smirnov test. The differences in analytes between participants in Tocovid and placebo group were tested using a Chi-Square test or Fisher’s Exact test depending on suitability of categorical variables, whereas an independent t-test or Mann–Whitney U test was performed for continuous variables depending on normality of data. *p*-values of <0.05 were deemed statistically significant.

## 3. Results

### 3.1. Validation of Nerve Conduction Study Methodology

Prior to the commencement of the randomized controlled trial, validation of the NCS methodology was conducted by comparing diabetic participants to age-matched volunteers without diabetes mellitus. It was apparent that the nerve conduction parameters for all nerves tested among the normal age-matched volunteers were significantly higher than the diabetic participants, and all the results were highly significant (*p* < 0.001). The results are depicted in Table 1.

### 3.2. Randomized Controlled Trial

**Figure 1:**
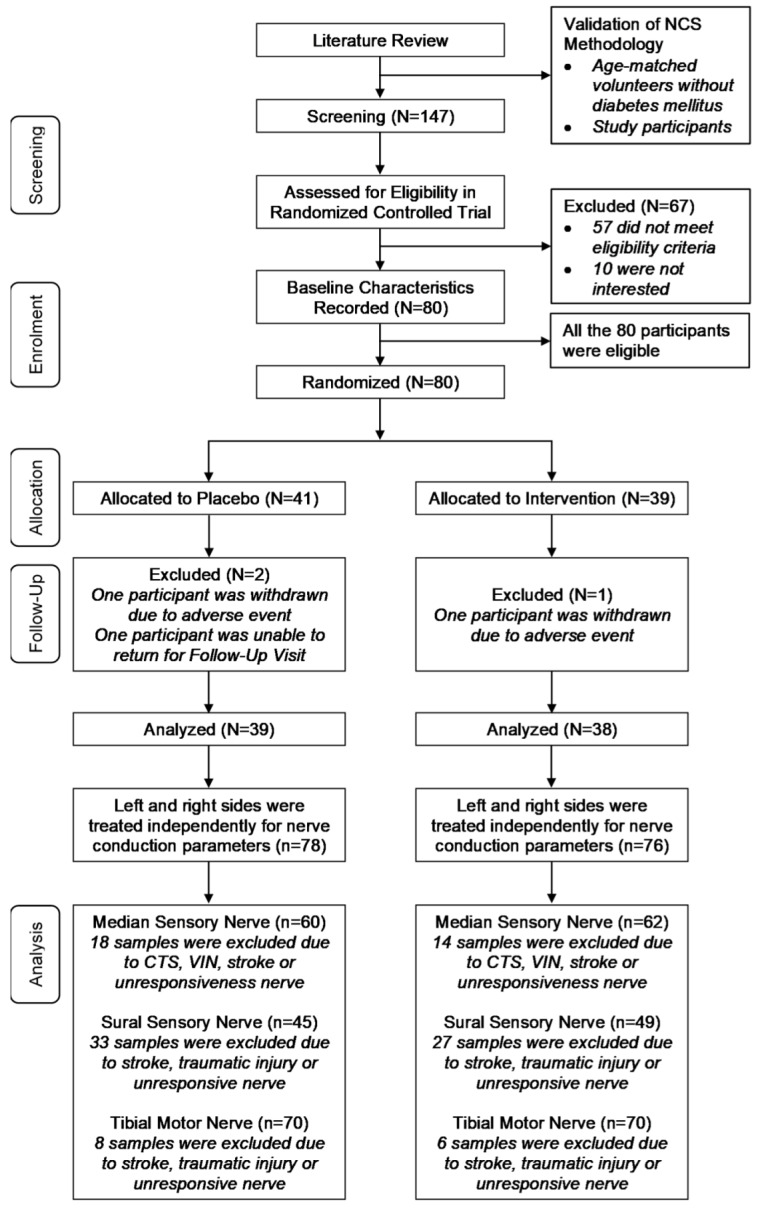
Study design flow chart. N: Number of participants; *n*: Sample size for nerve conduction parameters; CTS: Carpal tunnel syndrome; VIN: Vibration-induced neuropathy.

A total of 147 patients with T2DM were screened over the period of two months. Out of these patients, 80 participants were recruited for the clinical trial (number of participants (percentage) sex, 52 (65.0%) male; median (IQR) age, 63.50 [14.00] years; median (IQR) duration of diabetes, 14.00 (10.00) years; median (IQR) HbA1c, 7.40 (1.70) %). However, only 77 of the 80 participants completed the randomized controlled trial. Among the participants who completed the study, 38 subjects were in the tocotrienol group, whereas 39 subjects were in the placebo group. The left and right sides of each nerve measured were treated independently. The nerves which were affected by conditions other than diabetes mellitus and nerves which were unresponsive upon stimulation were excluded. As a result, the sample sizes were 122, 94 and 140 for the median, sural and tibial nerves, respectively. A summary of the study design is depicted in Figure 1.

The baseline characteristics of the participants recruited are illustrated in Table 2. There were no significant differences in gender, ethnicity, age, duration of diabetes, HbA1c, blood pressure, BMI, serum biomarkers (NGF, MDA, TNFR-1, VCAM-1, TXB2), and safety tests (renal profile, liver function test, lipid profile) between the Tocovid and placebo groups at baseline.

The nerve conduction parameters for median, sural and tibial nerves were measured bilaterally for all 80 eligible participants. Three participants who did not complete the trial were not analyzed as per protocol analyses were performed for this clinical trial. At baseline, all the nerve conduction parameters evaluated for the median, sural and tibial nerves were not statistically significant between the Tocovid and placebo groups. At the end of the trial, the mean and median of change between all the nerve conduction parameters were calculated and compared between the Tocovid and placebo groups (Table 3). It was found that all the velocities parameters in every nerve significantly increased among the Tocovid group as compared to the placebo group after eight weeks of supplementation. Among participants in the Tocovid group, the conduction velocity increased by 1.25 m/s (IQR 3.35, *p* < 0.001), 1.60 m/s (IQR 1.80, *p* < 0.001), and 0.75 m/s (IQR 2.25, *p* < 0.001) for the median, sural, and tibial nerves respectively; the peak velocity increased by 0.95 m/s (IQR 2.40, *p* < 0.001) and 1.14 m/s (SD 1.64, *p* < 0.001) for the median and sural nerves, respectively. Nevertheless, the amplitude of action potential for all nerves was not statistically different between the Tocovid and placebo groups.

Serum NGF levels were significantly higher among the participants given Tocovid compared to placebo (*p* = 0.047) after eight weeks of supplementation (Table 4). Other serum biomarkers including MDA, VCAM-1, TNFR-1, and TXB2 were not statistically different between Tocovid and placebo groups at the end of eight weeks. Besides, HbA1c, blood pressure, BMI, renal profile and liver function tests remained similar and were not statistically different between both groups at the end of the study. Total cholesterol was slightly higher among participants in the Tocovid group compared to placebo (*p* = 0.036) after eight weeks of intervention. Meanwhile, HDL-C levels were similar between the Tocovid and placebo groups (*p* = 0.569) at eight weeks post-intervention.

## 4. Discussion

Vitamin E, in particular its tocotrienol isoform, exhibits potent antioxidant and anti-inflammatory properties [9]. These properties of Vitamin E are well established and stem from the ability of the hydroxyl group in the chromanol ring to stabilize the reactive oxygen species (ROS) and other free radicals by donating a hydrogen ion [9]. Laboratory researches done in the past indicated that the quantity of methyl groups on the Vitamin E will determine its antioxidant effects [9]. In addition, it was demonstrated that the α-, β-, γ-isoforms of both tocotrienol and tocopherol are similar in terms of antioxidant property; whereas the δ-isoforms of Vitamin E have the weakest antioxidant effect [9,25].

Even though similar properties have been shown between tocotrienols and tocopherols in laboratory studies, tocotrienol isoforms are more superior than their tocopherol counterparts in vivo. Tocotrienols isoforms have been shown to be more superior than α-tocopherol in decreasing lipid peroxidation and peroxyl radicals in liposomal membranes and rat liver [26,27,28], reducing reactive substances in endothelial cells of human umbilical veins [29], decreasing oxidative damage in rat brains [30], as well as reducing the risk of oxidative stress in nematodes [31]. These can be explained by the molecular structure and function of tocotrienol. First and foremost, there is a more consistent distribution of tocotrienol in cellular plasma membranes [9]. Besides, it has a higher disordering effect than tocopherol due to the presence of double bonds, giving it a better interaction with the free radicals produced [9]. Lastly, the chromanoxyl radicals in Tocotrienol also have higher recycling properties compared to tocopherol [9].

Thus, tocotrienol-rich vitamin E was believed to be beneficial among patients with DPN, but the evidence is scarce. Based on a clinical trial published by Tutuncu NB et al. two decades ago, daily oral administration of vitamin E for six months improved median motor nerve conduction velocity and tibial motor nerve distal latency [11]. Otherwise, there were no studies which reported the objective improvement of nerve functions as assessed by nerve conduction parameters among diabetic patients after vitamin E administration. Besides, there were studies which illustrated that vitamin E could potentially alleviate the symptoms of neuropathic pain among patients with DPN as assessed by self-reported questionnaires [10,12]. However, the use of this method of assessment may be deemed subjective. Despite the promising results reported from the aforementioned studies [10,11,12], it is important to note that all of these studies did not specify the actual proportion of tocotrienol in the vitamin E. In addition, it was previously thought that α-tocopherol was the most superior isoform of vitamin E, and up to the year 2015, only approximately 3% of the published literature regarding vitamin E were concerning tocotrienol [9].

In this randomized placebo-controlled trial, it was clearly shown that oral administration of tocotrienol-rich vitamin E for eight weeks significantly improved the velocities (CV and PV) of all nerves but not the amplitude of action potentials. These results were contrary to a recent similar study conducted by Hor CP et al. which concluded that the oral administration of tocotrienol-rich vitamin E for a year did not improve sensory nerve conduction parameters [13]. Additionally, as the study recruited participants who were symptomatic for DPN, it was also illustrated that there were no beneficial effects in relieving DPN symptoms as assessed by Total Symptom Score (TSS) and Neuropathy Impairment Score (NIS). The contradictory findings from this clinical trial may be attributable to the criteria for recruitment in which participants were eligible regardless of whether they experienced any symptoms of DPN.

The highly significant improvement in terms of the velocities in all nerves demonstrated in this study can be explained by firstly understanding the mechanism behind nerve conduction parameter changes among diabetic patients. According to a study conducted by Valls-Canals J et al., early in the course of diabetes, when there are still no clinical symptoms of DPN, evidence of demyelination has already been shown by reduced nerve conduction velocity compared to individuals without diabetes upon electrophysiological testing [32]. As diabetes progresses and patients develop clinical symptoms of DPN, both demyelination and axonal degeneration were seen as reflected by the reduction in conduction velocity and decreased amplitude of action potential, respectively, upon NCS testing [32]. With reference to our randomized controlled trial, we thus postulated that only in the early stages of DPN before clinical symptoms develop, progression of DPN could be reversed by intervention, specifically tocotrienol-rich vitamin E in our clinical trial. This clearly explains the improvements—only in velocity parameters of nerve conduction, but not in amplitude of action potential—after eight weeks of Tocovid supplementation.

Based on the oxidative stress pathogenesis of DPN, chronic hyperglycemia causes excessive production of superoxide, a precursor of reactive oxygen species (ROS) [33], in the mitochondria [34]. The superoxide will then be converted to hydrogen peroxide and other ROS which will cause damage to the cellular tissues in diabetic patients, leading to diabetic complications, particularly DPN [34]. Besides, the superoxide overproduction in neuronal tissues will also contribute to neuronal dysfunction by activating the formation of AGEs, polyol pathways, hexosamine pathway, PKC pathway and several other metabolic pathways [35]. With regard to cellular damage, plasma membranes containing lipids are most susceptible to injury by ROS, by means of lipid peroxidation [36]. As the major biochemical structure of the neuronal myelin sheath is made up of lipids (70–80% lipids, 20–30% proteins) [37], a marker of lipid peroxidation product, MDA, which was significantly increased in patients with diabetic neuropathy compared to normal healthy adults [38], was measured in our clinical trial to investigate the antioxidative effects of Tocovid. We hypothesized that the antioxidative properties of tocotrienol-rich vitamin E will improve DPN by inhibiting the superoxide production, reducing lipid peroxidation.

Apart from oxidative stress, chronic inflammation is another main feature in diabetes mellitus which contributes to the development of DPN based on current perspectives [39]. High blood glucose triggers the proinflammatory pathways which are interconnected with the other metabolic pathways mentioned above [39]. For instance, ROS produced from the oxidative stress pathway will stimulate several proinflammatory pathways [34]; AGE interaction with receptors for AGEs (RAGE) will in turn activate transcription factor, nuclear factor-κβ (NF-κβ), inducing a proinflammatory gene expression, producing cytokines such as tumor necrosis factor-α (TNF-α) and interleukin (IL)-1β [40]. TNFR-1 is one of the receptors for TNF-α, and the binding of TNF-α to TNFR-1 activates the inflammatory pathway [41], which plays a role in development of diabetic complications [42]. Other than that, chronic inflammation in patients with diabetes mellitus demonstrated increased circulating vascular cell adhesion molecule-1 (VCAM-1) and intracellular adhesion molecule-1 (ICAM-1) [43,44]. All these molecules are associated with the development of DPN. As a potent anti-inflammatory agent, tocotrienol-rich vitamin E had been expected to reduce these proinflammatory cytokines. In this clinical trial, VCAM-1 and TNFR-1 were measured to investigate the anti-inflammatory properties of Tocovid on DPN.

However, serum biomarkers reflecting oxidative stress (MDA) and inflammatory activities (VCAM-1 and TNFR-1) remained similar between both Tocovid and placebo groups at the end of this clinical trial, suggesting possible alternative pathways targeted by Tocovid in enhancing nerve conduction velocities. With respect to the alternative pathways which correspond to the improved nerve velocity parameters, it was apparent from this randomized placebo-controlled clinical trial that the neuronal cell biomarkers, serum NGF, appeared to be significantly elevated among the participants on Tocovid supplementation compared to placebo at eight weeks post-intervention. This increase in serum NGF reflects reversal of neuronal injuries as well as the restoration of nerve function [45], and it is a novel finding, as no studies in the past have investigated the effects of tocotrienol-rich vitamin E on serum NGF and correlated it to improvements in terms of peripheral nerve conduction velocities.

The safety tests done in this clinical trial illustrated that Tocovid significantly increased the total cholesterol levels after eight weeks of supplementation compared to placebo, but the levels were deemed not clinically significant as they were below the upper limit of normal values (5.18 mmol/L). Otherwise, other safety parameters were not statistically significant between both groups.

## 5. Conclusions

All in all, this is the first clinical trial to illustrate that tocotrienol-rich vitamin E (Tocovid) supplementation for eight weeks is able to improve DPN as assessed by nerve conduction velocity improvements in median sensory, sural sensory and tibial motor nerves. These improvements also correlate with elevation in serum NGF levels after eight weeks of supplementation with Tocovid compared to placebo. Based on this clinical trial, Tocovid most probably acts through the pathway involving NGF—and not the oxidative stress or chronic inflammatory pathogeneses—to improve DPN. Nonetheless, further studies should affirm the definite pharmacodynamics of tocotrienol-rich vitamin E in order to include Tocovid in the treatment regime for medical conditions with similar pathophysiological mechanisms to DPN.

## Figures and Tables

**Table 1:** Age-matched comparison of nerve conduction parameters in median, sural and tibial nerves between study subjects and normal controls.

Nerve Conduction Parameter	Study Subjects	Normal Controls	*p*-Value
Median Sensory Nerve	*n* = 20 ^‡^	*n* = 20 ^§^	
Conduction Velocity (m/s) ^a^	42.84 ± 5.80	53.47 ± 4.51	<0.001 *
Peak Velocity (m/s) ^a^	34.21 ± 4.02	41.65 ± 3.68	<0.001 *
NP Amplitude (µV) ^b^	18.40 (8.15)	31.20 (18.60)	<0.001 *
Sural Sensory Nerve	*n* = 24 ^†^	*n* = 24 ^†^	
Conduction Velocity (m/s) ^b^	42.75 (4.93)	50.45 (4.50)	<0.001 *
Peak Velocity (m/s) ^a^	33.56 ± 2.90	39.18 ± 3.22	<0.001 *
PP Amplitude (µV) ^a^	7.12 ± 4.87	13.65 ± 5.35	<0.001 *
Tibial Motor Nerve	*n* = 24 ^†^	*n* = 24 ^†^	
Conduction Velocity (m/s) ^b^	41.90 (8.17)	49.55 (3.90)	<0.001 *
Distal Amplitude at Ankle (mV) ^a^	7.65 ± 3.62	12.69 ± 3.50	<0.001 *

*n*: Sample size; NP: Negative peak; PP: Positive peak. ^†^ Twelve study subjects were compared to twelve age-matched normal controls, yielded *n* = 24 for each group as left and right were treated independently. ^‡^ *n* = 4 were excluded due to carpal tunnel syndrome. ^§^ *n* = 3 were excluded due to carpal tunnel syndrome; *n* = 1 was excluded due to ganglion cyst on wrist. * Data are significant if *p* < 0.05. ^a^ Data presented as mean ± standard deviation; *p*-value obtained using independent t-test, assumptions were fulfilled. ^b^ Data presented as median (interquartile range); *p*-value obtained using Mann–Whitney U test.

**Table 2:** Baseline characteristics of study subjects.

Baseline Characteristic	Placebo (*n* = 41)	Tocovid (*n* = 39)	*p*-Value	Total (*n* = 80)
Gender, n (%)			0.870 ^†^	
Male	27 (65.9)	25 (64.1)		52 (65.0)
Female	14 (34.1)	14 (35.9)		28 (35.0)
Race, n (%)			0.808 ^‡^	
Malay	19 (46.3)	20 (51.3)		39 (48.8)
Chinese	12 (29.3)	10 (25.6)		22 (27.5)
Indian	7 (17.1)	8 (20.5)		15 (18.8)
Others	3 (7.3)	1 (2.6)		4 (5.0)
Age (years) ^b^	64.00 (15.00)	63.00 (12.00)	0.836	63.50 (14.00)
Duration of DM (years) ^b^	13.00 (11.00)	14.00 (10.00)	0.916	14.00 (10.00)
HbA1c (%) ^b^	7.40 (1.85)	7.20 (1.70)	0.509	7.40 (1.70)
SBP (mmHg) ^a^	129.39 ± 12.99	133.90 ± 12.76	0.122	131.59 ± 13.00
DBP (mmHg) ^a^	77.90 ± 9.12	78.08 ± 8.83	0.931	77.99 ± 8.92
BMI (kg/m^2^) ^a^	28.36 ± 5.07	27.99 ± 4.17	0.722	28.18 ± 4.63
Serum Biomarkers				
NGF (ng/mL) ^b^	11.47 (4.11)	11.11 (4.58)	0.59	11.26 (3.62)
MDA (ng/mL) ^b^	881.03 (697.85)	794.22 (726.90)	0.881	878.54 (713.99)
VCAM-1 (ng/mL) ^b^	347.15 (311.90)	412.47 (275.07)	0.438	382.40 (274.14)
TNFR-1 (pg/mL) ^b^	66.87 (56.64)	80.37 (63.71)	0.22	71.54 (59.51)
TXB2 (pg/mL) ^b^	83.96 (73.01)	90.11 (61.67)	0.634	85.78 (67.90)
α-Tocopherol (µmol/L)	40.46 ± 18.73	42.05 ± 25.72	0.753	41.23 ± 22.23
Safety Tests				
eGFR ^a^	64.34 ± 21.92	67.23 ± 20.86	0.548	65.75 ± 21.33
Urea ^b^	6.15 (3.63)	5.65 (2.60)	0.606	5.75 (3.30)
AST ^b^	20.00 (9.50)	20.00 (11.00)	0.928	20.00 (10.00)
ALT ^b^	21.00 (15.50)	23.00 (17.50)	0.976	21.00 (17.00)
TC ^a^	4.56 ± 0.98	4.91 ± 1.11	0.14	4.73 ± 1.05
HDL-C ^b^	1.20 (0.48)	1.30 (0.40)	0.37	1.30 (0.40)

*n*: Number of participants; n: Number of participants in subgroup; DM: Diabetes mellitus; HbA1c: Hemoglobin A1c; SBP: Systolic blood pressure; DBP: Diastolic blood pressure; BMI: Body mass index; NGF: Nerve growth factor; MDA: Malondialdehyde; VCAM-1: Vascular cell adhesion molecule-1; TNFR-1: Tumor necrosis factor receptor-1; TXB2: Thromboxane B2; α-Tocopherol: Corrected serum α-tocopherol levels; eGFR (ml/min/1.73 m^2^): Estimated glomerular filtration rate; Urea (mmol/L); AST (U/L): Aspartate transaminase; ALT (U/L): Alanine transaminase; TC (mmol/L): Total cholesterol; HDL-C (mmol/L): High-density lipoprotein cholesterol. * Data are significant if *p* < 0.05. ^a^ Data presented as mean ± standard deviation; *p*-value obtained using independent t-test, assumptions were fulfilled. ^b^ Data presented as median (interquartile range); *p*-value obtained using Mann–Whitney U test. ^†^ Chi-Square Test. Assumptions were fulfilled. ^‡^ Fisher’s Exact Test. More than 20% of the cells have expected value of less than 5.

**Table 3:** Comparison of nerve conduction parameters between the Tocovid and placebo groups.

**Median Sensory Nerve ^†^**	**Placebo (*n* = 60)**	**Tocovid (*n* = 62)**	***p*-Value**
Conduction Velocity (m/s)			
At Baseline ^b^	44.10 (9.07)	43.45 (10.43)	0.838
At Eight Weeks ^a^	43.44 ± 5.94	46.29 ± 6.65	0.014 *
Change ^b^	0.00 (2.90)	1.25 (3.35)	<0.001 *
Peak Velocity (m/s)			
At Baseline ^a^	35.21 ± 4.86	35.45 ± 4.91	0.778
At Eight Weeks ^b^	35.40 (5.65)	35.40 (8.55)	0.218
Change ^b^	−0.15 (2.33)	0.95 (2.40)	<0.001 *
NP Amplitude (µV)			
At Baseline ^b^	17.80 (11.93)	20.55 (14.25)	0.259
At Eight Weeks ^b^	17.75 (13.65)	22.35 (15.60)	0.102
Change ^a^	1.15 ± 4.93	1.70 ± 5.95	0.58
PP Amplitude (µV)			
At Baseline ^b^	28.95 (17.78)	32.15 (19.70)	0.251
At Eight Weeks ^b^	29.25 (20.28)	34.90 (21.78)	0.032 *
Change ^b^	1.40 (8.30)	3.15 (11.35)	0.213
**Sural Sensory Nerve** ^‡^	**Placebo (*n* = 45)**	**Tocovid (*n* = 49)**	***p*-Value**
Conduction Velocity (m/s)			
At Baseline ^a^	43.15 ± 5.53	43.53 ± 5.92	0.751
At Eight Weeks ^a^	42.29 ± 4.51	45.32 ± 5.30	0.004 *
Change ^b^	−0.60 (2.10)	1.60 (1.80)	<0.001 *
Peak Velocity (m/s)			
At Baseline ^a^	34.36 ± 3.73	34.32 ± 4.49	0.955
At Eight Weeks ^b^	33.30 (3.70)	35.00 (7.65)	0.133
Change ^a^	−0.54 ± 1.62	1.14 ± 1.64	<0.001 *
PP Amplitude (µV)			
At Baseline ^b^	8.70 (8.05)	7.00 (8.45)	0.5
At Eight Weeks ^b^	7.40 (8.60)	7.70 (11.35)	0.904
Change ^a^	0.43 ± 2.99	1.11 ± 2.57	0.243
NP Amplitude (µV)			
At Baseline ^b^	10.10 (5.50)	9.80 (9.40)	0.498
At Eight Weeks ^b^	8.50 (5.60)	9.20 (9.25)	0.31
Change ^b^	−0.40 (2.10)	0.10 (4.95)	0.47
**Tibial Motor Nerve ^§^**	**Placebo (*n* = 70)**	**Tocovid (*n* = 70)**	***p*-Value**
Conduction Velocity (m/s)			
At Baseline ^a^	40.17 ± 6.14	41.44 ± 5.69	0.206
At Eight Weeks^a^	39.02 ± 5.79	42.40 ± 4.89	<0.001 *
Change ^b^	−0.90 (3.50)	0.75 (2.25)	<0.001 *
Distal Amplitude at Ankle (mV)			
At Baseline ^a^	7.87 ± 4.78	8.32 ± 4.08	0.547
At Eight Weeks ^a^	8.78 ± 5.07	9.44 ± 4.12	0.398
Change ^b^	0.60 (1.83)	0.80 (1.95)	0.291

*n*: Sample size; Change: Nerve conduction parameter at eight weeks minus baseline; NP: Negative peak; PP: Positive peak. ^†^*n* = 38 were excluded for analyses: 15 with carpal tunnel syndrome, 4 with vibration-induced neuropathy, 5 were affected with stroke, 8 had unresponsive nerve upon stimulation; 6 were withdrawn from the study. ^‡^ *n* = 66 were excluded for analyses: 5 were affected with stroke, 2 had traumatic injury resulting in disrupted anatomy, 4 were unreliable due to obesity, 1 was unreliable due to gouty tophi at lateral malleolus, 48 had unresponsive nerve upon stimulation, 6 were withdrawn from the study. ^§^ *n* = 20 were excluded for analyses: 5 were affected with stroke, 1 had traumatic injury resulting in disrupted anatomy, 8 had unresponsive nerve upon stimulation, 6 were withdrawn from the study. * Data are significant if *p* < 0.05. ^a^ Data presented as mean ± standard deviation; *p*-value obtained using independent t-test, assumptions were fulfilled. ^b^ Data presented as median (interquartile range); *p*-value obtained using Mann–Whitney U test.

**Table 4:** End of eight weeks comparison of analytes between Tocovid and placebo groups.

Analyte	Placebo (*n* = 39)	Tocovid (*n* = 38)	*p*-Value
Serum Biomarkers			
NGF (ng/mL) ^a^	10.02 ± 2.28	11.04 ± 2.05	0.047 *
MDA (ng/mL) ^b^	1158.37 (794.77)	959.57 (903.02)	0.551
VCAM-1 (ng/mL) ^a^	257.40 ± 113.23	278.28 ± 129.36	0.453
TNFR-1 (pg/mL) ^b^	23.24 (55.52)	21.88 (45.43)	0.874
TXB2 (pg/mL) ^b^	162.57 (164.41)	150.70 (132.24)	0.418
HbA1c (%) ^a^	7.81 ± 1.29	7.43 ± 1.11	0.174
SBP (mmHg) ^a^	129.00 ± 13.95	133.16 ± 14.70	0.207
DBP (mmHg) ^b^	75.00 (14.00)	73.00 (16.00)	0.386
BMI (kg/m^2^) ^a^	27.81 ± 4.89	27.57 ± 4.29	0.823
α-Tocopherol (µmol/L)	37.90 ± 17.61	67.10 ± 38.59	<0.001 *
Safety Tests			
eGFR ^b^	62.00 (39.00)	63.50 (42.00)	0.303
Urea ^a^	6.90 ± 2.61	6.63 ± 2.47	0.651
AST ^b^	19.00 (8.00)	18.50 (8.25)	0.394
ALT ^b^	20.00 (12.00)	19.00 (14.00)	0.298
TC ^a^	4.43 ± 1.10	4.95 ± 1.03	0.036 *
HDL-C ^b^	1.20 (0.30)	1.30 (0.50)	0.569

*n*: Number of participants in subgroup; NGF: Nerve growth factor; MDA: Malondialdehyde; VCAM-1: Vascular cell adhesion molecule-1; TNFR-1: Tumor necrosis factor receptor-1; TXB2: Thromboxane B2; HbA1c: Hemoglobin A1c; SBP: Systolic blood pressure; DBP: Diastolic blood pressure; BMI: Body mass index; α-Tocopherol: Corrected serum α-tocopherol levels; eGFR (ml/min/1.73 m^2^): Estimated glomerular filtration rate; Urea (mmol/L); AST (U/L): Aspartate transaminase; ALT (U/L): Alanine transaminase; TC (mmol/L): Total cholesterol; HDL-C (mmol/L): High-density lipoprotein cholesterol. * Data are significant if *p* < 0.05. ^a^ Data presented as mean ± standard deviation; p-value obtained using independent t-test, assumptions were fulfilled. ^b^ Data presented as median (interquartile range); *p*-value obtained using Mann–Whitney U test.

## Supplementary Materials

The following are available online at https://www.mdpi.com/2072-6643/12/5/1522/s1, The contents of Tocovid, details of NCS methodology used and participating investigators are available online, Constituents of Tocovid, Detailed Procedure of Nerve Conduction Study, List of Participating Investigators.
