# Supplementary material for: The Effects of Tocotrienol-Rich Vitamin E (Tocovid) on Diabetic Neuropathy: A Phase II Randomized Controlled Trial
Source: Nutrients. 2020 May 23;12(5):1522. doi: 10.3390/nu12051522 (PMC7284602; doi:10.3390/nu12051522)
Supplement: Supplementary file 1 [file nutrients-12-01522-s001.zip › Nutrients Constituents of Tocovid.docx]

Constituents of Tocovid

*Tocotrienols contents:*

200mg

*Ingredients:*

1. d-α-Tocotrienol: 61.52mg
2. d-γ-Tocotrienol: 112.80mg
3. d-δ-Tocotrienol: 25.68mg
4. d-α-Tocopherol: 91.60IU
5. Plant Squalene: 51.28mg
6. Phytosterol Complex: 20.48mg
