# Supplementary material for: The Effects of Tocotrienol-Rich Vitamin E (Tocovid) on Diabetic Neuropathy: A Phase II Randomized Controlled Trial
Source: Nutrients. 2020 May 23;12(5):1522. doi: 10.3390/nu12051522 (PMC7284602; doi:10.3390/nu12051522)
Supplement: Supplementary file 1 [file nutrients-12-01522-s001.zip › Nutrients Detailed Procedure of Nerve Conduction Study.docx]

Detailed Procedure of Nerve Conduction Study

*Sensory Nerve Conduction Study*

Sensory NCS for both the median and sural nerves were performed using the antidromic technique (stimulator placed proximally and recording electrode placed distally, i.e. action potential recorded transverse the nerve in a physiologically opposite direction). A pair of recording electrodes (G1 and G2) were placed along the nerve being studied, with the active electrode (G1) placed closer to the cathode of the stimulator, and the distance between G1 and G2 was ensured to be approximately 3cm to 4cm apart. For instance, when performing median sensory NCS, the recording electrodes were placed on the palmar surface of the hand, with G1 electrode placed slightly distal to the second metacarpophalangeal joint and G2 electrode placed slightly proximal to the second distal interphalangeal joint; when performing sural sensory NCS, the G1 electrode was placed posterior to the lateral malleolus and G2 electrode placed 3cm distal to the G1 electrode. The cathode of the stimulating electrode was placed 14cm away from G1 electrode for both the median and sural sensory NCS; the stimulating electrode was placed between the flexor carpi radialis and the palmaris longus tendons in median sensory NCS, while the stimulating electrode was placed slightly lateral to the calf midline for sural sensory NCS.

Filter settings were set at 10Hz to 5kHz, sweep speed was 1ms per division, gain was 20µV per division and duration of stimulation was 200µs. A current of between 5mA to 30mA was used to achieve supramaximal electrical stimulation. The parameters recorded in sensory NCS include onset latency, peak latency, sensory nerve action potential (SNAP), conduction velocity (CV), and peak velocity (PV).

*Motor Nerve Conduction Study*

Meanwhile, motor NCS were recorded by the orthodromic technique (action potential recorded transverse the nerve in the same direction as the physiological nerve impulse conduction). The recording electrodes were positioned using the belly-tendon montage. For tibial nerve motor NCS, the G1 electrode was placed over the abductor hallucis muscle belly (slightly anteroinferior to the navicular tubercle), whereas the G2 electrode was placed over the tendon (medial aspect of the first metatarsophalangeal joint). Due to the presence of neuromuscular junction in motor nerve fibers, two sites along a motor nerve need to be stimulated in order to obtain an accurate result for the parameters in motor NCS. For instance, when carrying out the tibial motor NCS in this study, the cathode of the stimulator was first positioned posterior to the medial malleolus (8cm from the G1 electrode), and subsequently positioned at the popliteal fossa. The distance between the two points (distal and proximal points of stimulation) was recorded for the motor NCS parameters calculation.

Filter settings were set at 3Hz to 10kHz, sweep speed was 5ms per division, gain was 5mV per division and duration of stimulation was 200µs. A current of between 20mA to 100mA was used to achieve supramaximal electrical stimulation. The parameters recorded in motor NCS include distal and proximal onset latencies as well as compound muscle action potential (CMAP) respectively. In addition, the distance between the distal and proximal stimulation point were recorded along with the conduction velocity (CV).
