# Supplementary material for: The Effects of Tocotrienol-Rich Vitamin E (Tocovid) on Diabetic Neuropathy: A Phase II Randomized Controlled Trial
Source: Nutrients. 2020 May 23;12(5):1522. doi: 10.3390/nu12051522 (PMC7284602; doi:10.3390/nu12051522)
Supplement: Supplementary file 1 [file nutrients-12-01522-s001.zip › Nutrients List of Participating Investigators.docx]

List of Participating Investigators

- Ng Yeek Tat^1,2^
- Sonia Phang Chew Wen^1^
- Gerald Tan Chen Jie^1,2^
- Ng En Yng^1,2^
- Nevein Philip Botross Henien^2^
- Uma Devi M Palanisamy^1^
- Badariah Ahmad^1^
- Khalid Abdul Kadir^1,2,3^

*Recruitment and Research Centers*

^1^Monash University Clinical Research Center Bandar Sunway

^2^Monash University Clinical Research Center Johor Bahru

^3^Thomson Hospital Kota Damansara
